# Kidney Biopsy in Patients With Markedly Reduced Kidney Function

**DOI:** 10.1016/j.ekir.2022.08.004

**Published:** 2022-08-17

**Authors:** Mohamad M. Alkadi, Essa A. Abuhelaiqa, Shaefiq B. Thappy, Fatima B. Eltayeb, Khaled A. Murshed, Mohammed Akhtar, Omran I. Almokdad, Hassan A. Al-Malki, Abdullah I. Hamad, Ahmed F. Hamdi, Omar M. Fituri, Adel M. Ashour, Awais Nauman, Hiba Tohid, Rajvir Singh, Muhammad Asim

**Affiliations:** 1 Division of Nephrology, Department of Medicine, Hamad Medical Corporation, Doha, Qatar; 2 Weill Cornell Medical College—Qatar, Doha, Qatar; 3 Division of Anatomic Pathology, Department of Laboratory Medicine and Pathology, Hamad Medical Corporation, Doha, Qatar; 4 Division of Interventional Radiology, Department of Clinical Imaging, Hamad Medical Corporation, Doha, Qatar; 5 Department of Biostatistics, Hamad Medical Corporation, Doha, Qatar

**Keywords:** acute kidney injury, chronic kidney disease, complications, kidney biopsy, outcomes, predictors

## Introduction

One of the challenges physicians face while evaluating patients with markedly reduced kidney function is distinguishing between acute kidney injury and chronic kidney disease (CKD). The coexistence of more than one disorder is well recognized as CKD is a major risk factor for acute kidney injury, from which the patient may not recover completely, leading to accelerated renal dysfunction.^1^ Determining baseline kidney function by retrospectively analyzing serum creatinine values over an extended period is essential in such cases. Nevertheless, the usefulness of kidney biopsy in advanced CKD is often questioned due to the low therapeutic yield and high risks.^2^^,^^3^^,^S1,S2 The primary objective of this study is to assess the benefits and risks associated with kidney biopsy in patients presenting with an estimated glomerular filtration rate (eGFR) of <15 ml/min per 1.73 m^2^ when previous kidney function is unknown and immunology screening result is not indicative of a systemic disorder. Secondary objectives include determining predictors of histologically severe CKD and risk factors for kidney biopsy complications.

## Results

**Figure 1:**
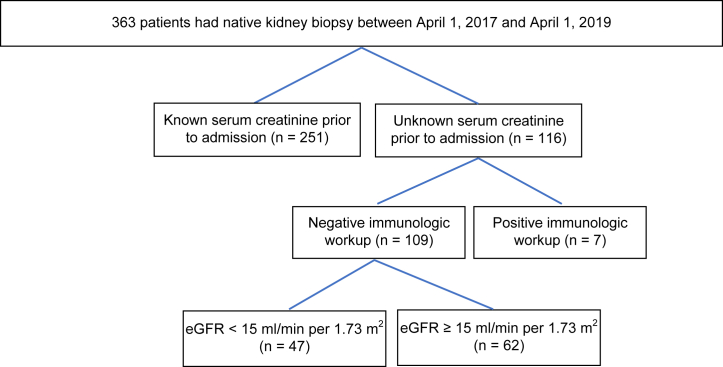
Flowchart of the study design. Of 363 patients who had native kidney biopsy during their hospitalization, 109 met the inclusion criteria of unknown baseline kidney function and negative immunologic workup result. Of the patients, 43% (*n* = 47) had eGFR < 15 ml/min per 1.73 m^2^. eGFR, estimated glomerular filtration rate.

Between April 1, 2017, and April 1, 2019, 363 patients were admitted and underwent native kidney biopsy; 30% (*n* = 109) had unknown baseline creatinine level and negative immunology screening result and were included in the study (Figure 1). Most patients were males and Asians. Their mean age was 35 ± 10.6 years. At the time of biopsy, 47 patients (43%) had eGFR < 15, 14 (13%) had eGFR 15 to 29, 18 (17%) had eGFR 30 to 59, and 30 (28%) had eGFR ≥ 60 ml/min per 1.73 m^2^. Patients with eGFR < 15 ml/min per 1.73 m^2^ were more likely than patients with higher eGFR to have inadequate kidney biopsy samples (34% vs. 11%; *P* = 0.004), nontreatable acute pathological findings (87% vs. 47%; *P* < 0.0001), and postbiopsy complications (28% vs. 2%; *P* < 0.0001). Patients’ demographics are summarized in Table 1.

**Table 1:** Baseline characteristics of study population

Variable	GFR < 15 (*n* = 47)	GFR ≥ 15 (*n* = 62)	*P* value
Creatinine at biopsy, μmol/l	813 (593–1260)	140 (79–222)	<0.0001
eGFR at biopsy, ml/min per 1.73 m^2^	8 (7–10)	52 (31–104)	<0.0001
Age, yr	33 (30–44)	33 (27–39)	0.07
Male gender, *n* (%)	33 (70)	55 (89)	0.02
Race, *n* (%):			0.71
Middle Eastern	4 (9)	6 (10)	
Asian	37 (79)	52 (84)	
African	5 (11)	3 (5)	
Caucasian	1 (2)	1 (2)	
History of diabetes, *n* (%)	6 (13)	9 (15)	0.07
History of hypertension, *n* (%)	24 (51)	12 (19)	<0.001
SBP on presentation, mm Hg	170 (154–189)	148 (129–176)	0.02
DBP on presentation, mm Hg	105 (92–112)	88 (80–110)	0.09
UPC, mg/mmol, *n* (%)	583 (347–754)	359 (135–635)	0.03
Proteinuria > 3.5 g, *n* (%)	35 (74)	32 (52)	0.02
Hematuria, *n* (%)	30 (64)	38 (61)	0.07
Hemoglobin, g/dl	9 (8.1–9.8)	13 (11.6–14.7)	<0.0001
Calcium, mmol/l	2 (2.2–2.4)	2 (2.3–2.5)	<0.0001
Phosphorus, mmol/l	2 (1.7–2.3)	1 (1–1.4)	<0.0001
PTH, pg/ml	406 (274–661)	95 (48–179)	<0.0001
Albumin, g/l	26 (23–29)	24(13–28)	0.001
C3, mg/dl	100 (84–115)	124 (106–150)	<0.0001
C4, mg/dl	33 (28–39)	34 (25–42)	0.43
Kidney length, mm	97 (92–102)	105 (99–111)	<0.0001
Cortical thickness, mm	5 (5–7)	6 (5–7)	0.007
Parenchymal thickness, mm	14 (12–16)	14 (12–16)	0.34
Kidney hyperechogenicity, *n* (%)	47 (100)	46 (74)	<0.0001
Corticomedullary differentiation, *n* (%):			<0.0001
Maintained	3 (6)	30 (48)	
Poor	18 (38)	20 (32)	
Lost	26 (55)	12 (19)	
Adequacy of kidney biopsy, *n* (%)	31 (66)	55 (89)	0.004
Dialysis before kidney biopsy, *n* (%)	25 (53)	0	<0.0001
Biopsy complications, *n* (%)	13 (27)	1 (2)	<0.0001
Chronicity score, *n* (%):			<0.0001
Minimal	1 (2)	26 (42)	
Mild	1 (2)	9 (15)	
Moderate	23 (49)	20 (32)	
Severe	22 (47)	7 (11)	
Immunosuppression postbiopsy, *n* (%)	6 (13)	33 (53)	<0.0001

C3, complement factor 3; C4, complement factor 4; DBP, diastolic blood pressure; eGFR, glomerular filtration rate; PTH, parathyroid hormone; SBP, systolic blood pressure; UPC, urine protein-to-creatinine ratio.

Continuous variables are summarized as median (interquartile).

Of 47 patients with eGFR < 15 ml/min per 1.73 m^2^, 6 had acute renal pathological findings and got treated (13%). Acute interstitial nephritis was the most common finding, followed by immunoglobulin A nephropathy with crescents. Only 1 patient recovered kidney function after treatment (Supplementary Table S1). The remaining 41 patients had at least moderate to severe chronic changes on their biopsy samples based on their calculated chroncity score,S3 and 61% (*n* = 25) required hemodialysis initiation during admission. Immunoglobulin A nephropathy and hypertensive nephrosclerosis were the most identified causes (Supplementary Figure S1). Patients with chronicity scores < 8 were more likely to present with nausea and vomiting than patients with higher scores (48% vs. 9%; *P* = 0.005). Other variables were similar between both groups (Supplementary Table S2). Biopsy complications occurred in 28% of patients with eGFR < 15 ml/min per 1.73 m^2^ (hematoma without intervention [*n* = 6], hematoma requiring blood transfusion [*n* = 4], and hematoma requiring transfusion and embolization [*n* = 3]). The decision to transfuse was influenced by several factors, including clinical evidence of active bleeding, substantial drop in hemoglobin level postbiopsy, and the absolute level of postprocedure hemoglobin concentration, rather than the size of the hematoma. Patients who developed complications had lower baseline hemoglobin than patients with no complications (8.1 ± 1.1 vs. 9.2 ± 1.4; *P* = 0.02). The risk of hematoma was also higher in patients undergoing hemodialysis prebiopsy, but it did not reach significance (*P* = 0.06). All other variables were similar between both groups (Supplementary Table S3).

## Discussion

Kidney biopsy is the gold standard test that provides crucial information on the diagnosis, prognosis, and management of renal diseases. However, there is no universal consensus on the indications of renal biopsy.^2^ To our knowledge, this is the first study that discusses the benefits and risks associated with kidney biopsy in patients presenting with markedly reduced kidney function (eGFR < 15 ml/min per 1.73 m^2^), unknown baseline serum creatinine level, normal-sized kidneys, and negative immunology screening result. More than 95% of patients were found to have moderate to severe chronic changes on their kidney biopsy samples. Most patients were emigrants from Asia and were unaware of preexisting kidney disease. CKD in this population stratum is often not previously identified because either the CKD screening programs are not effectively implemented in their native countries^4^ or the public has inadequate access to health care due to economic barriers or lack of education/information. A study from a tertiary care center in Asia revealed that more than 50% of patients with CKD presented with stage 5 CKD.^5^

Predicting the degree of chronic histologic changes from clinical or diagnostic studies in patients with reduced kidney function continues to be a challenge. Besides nausea and vomiting, we did not find any specific clinical, laboratory, or radiologic findings correlating with renal histologic chronicity score. The overall incidence of postbiopsy hematoma was 28% in patients with eGFR < 15 ml/min per 1.73 m^2^ compared with <2% in patients with higher eGFR. Severe renal dysfunction poses a higher risk for bleeding because of (i) its association with scarred kidneys that are more likely to bleed and (ii) the coexistence of other risk factors, such as lower prebiopsy hemoglobin and higher urea and creatinine levels.^6^ In our study, a lower hemoglobin level at the biopsy was a statistically significant predictor of developing postbiopsy hematomas (*P* = 0.02). Anemia alters the flow of platelets in the bloodstream, decreasing the interaction between the platelets and the endothelium.^7^ It also reduces platelet aggregation by adenosine diphosphate, thromboxane, and nitric oxide pathways.^8^ The risk of hematoma was also higher in patients undergoing hemodialysis prebiopsy, but it did not reach significance (*P* = 0.06). Although dialysis therapy ameliorates platelet hemostatic dysfunction associated with uremia, there is evidence that interaction between the blood and the dialysis circuit can enhance bleeding, even in the absence of heparin, by temporarily worsening platelet aggregation and function.^9^ The requirement for dialysis also implies more severe kidney dysfunction and uremia-related abnormal platelet-platelet and platelet-endothelium interactions.

This study is limited by its retrospective nature and the number of patients, which might influence the statistical power. Furthermore, the decision to biopsy patients was determined by the nephrologist; hence, higher risk patients might not have been biopsied, a potential selection bias. Our study revealed that most patients with markedly reduced kidney function on presentation, unknown baseline creatinine level, normal-sized kidneys, and negative immunology screening result had advanced CKD with irreversible glomerular, tubulointerstitial, and vascular lesions, not amenable to treatment. Only 1 in 8 patients with an eGFR < 15 ml/min per 1.73 m^2^ on presentation had a treatable cause of the acute kidney injury. Of those, only 1 recovered kidney function. However, the rate of severe complications in that group exceeded 10%. Thus, discussing this risk-benefit ratio in the context of decision-making for a kidney biopsy in these patients would be necessary.

## Supplementary Materials

**Supplementary Methods.**

**Supplementary Results.**

**Supplementary References.**

**Figure S1.** Pie chart of the causes of advanced chronic kidney disease in patients with eGFR < 15 ml/min/1.73 m^2^. The etiology of advanced kidney disease was unknown in up to one-third of the cases. IgAN and hypertensive nephrosclerosis were the most identified causes of advanced chronic kidney disease.

**Table S1.** Clinical outcomes of patients with treatable acute kidney pathologic findings.

**Table S2.** Predictors of severe chronic changes in patients with eGFR < 15 ml/min per 1.73 m^2^.

**Table S3.** Kidney biopsy complications in patients with eGFR < 15 ml/min per 1.73 m^2^.
