# Supplementary material for: Kidney Biopsy in Patients With Markedly Reduced Kidney Function
Source: Kidney Int Rep. 2022 Aug 17;7(11):2505–8. doi: 10.1016/j.ekir.2022.08.004 (PMC9751579; doi:10.1016/j.ekir.2022.08.004)

SUPPLEMENTARY MATERIAL

1. SUPPLEMENTARY MATERIALS AND METHODS

1.1 Study Population:

In this retrospective cohort study, we reviewed patients admitted between April 1, 2017, and April 1, 2019, who underwent a kidney biopsy at Hamad Medical Corporation (HMC), Qatar's primary public healthcare provider. Patients younger than 18-year-old, kidney transplant recipients, patients with positive immunologic workup, and those with known prior-to-admission creatinine values got excluded from the study. The study design is summarized in Figure 1

1.2 Kidney biopsy protocol:

Kidney biopsy at our institution requires pre-biopsy blood pressure < 150/90 mm Hg, INR < 1.5, APTT< 1.2 X upper limit of normal range, and platelet count > 100 x 10^3 / ul. Antiplatelet medications are usually discontinued for at least 5 days before the biopsy. All biopsies are performed under ultrasound guidance by interventional radiologists. The biopsy is often taken from the lower pole of the left kidney using an 18-gauge biopsy needle, and two cores get sent for histology. Histopathology is interpreted by a renal pathologist using light microscopy and immunofluorescence. A post-biopsy Doppler ultrasound is performed to detect any hematomas. The patient is then kept in a supine position for 6 hours and monitored for gross hematuria, hemodynamic compromise, and significant persisting flank/abdominal pain. If the immediate post-biopsy color doppler US reveals a hematoma > 2 cm, the patient gets admitted to the

hospital and managed by a multi-disciplinary team of nephrologists, interventional radiologists, and urologists.

1.3 Data Collection:

Patients' clinical and laboratory data were collected from Cerner, a nationwide electronic medical record. Immunologic workup included complement levels (C3, C4), antinuclear antibodies, anti-double-stranded DNA antibodies, antineutrophil cytoplasmic antibodies, anti-glomerular basement membrane antibodies, and serum protein electrophoresis. The estimated Glomerular filtration rate (eGFR) was determined using the chronic kidney disease Epidemiology Collaboration (CKD-EPI) equation. We used serum creatinine level at the time of biopsy to calculate eGFR in patients who did not require renal replacement therapy to account for a correctable prerenal component. However, in patients who required renal replacement therapy before kidney, we used their serum creatinine level before initiation of dialysis to calculate eGFR. All renal ultrasound images were reviewed again by a radiologist. Kidney length was defined as the maximum longitudinal dimension. Parenchymal thickness was defined as the shortest distance from the renal sinus fat to the renal capsule, while cortical thickness was defined as the shortest distance from the base of a medullary pyramid to the renal capsule. Cortical echogenicity was graded relative to the liver parenchyma as Isoechogenic (equal to the liver) or hyperechogenic (higher than the liver). Corticomedullary differentiation was reported as maintained, poor, or lost. Two renal pathologists reviewed kidney biopsy slides. A kidney biopsy was considered adequate if it had at least 10 glomeruli for light microscopy and one glomerulus for immunofluorescence. A kidney biopsy chronicity score was determined based on the percentage of glomerulosclerosis, interstitial fibrosis, tubular atrophy, and arteriosclerosis[S3]. Patients were categorized into 4 groups according to their chronicity scores: minimal (0-1), mild

(2-4), moderate (5-7), and severe (≥ 8). A kidney biopsy complication was defined as any hematoma occurring post kidney biopsy regardless of its size. Post-biopsy hematomas that required a blood transfusion or an intervention such as embolization or nephrectomy were considered severe complications. This study was approved by our local institutional review board (MRC-01-20-798).

1.4 Statistical Analysis:

Data were summarized using frequencies with percentages measures for categorical variables and median with interquartile range for continuous variables. Unpaired t-test or Mann Whitney U test were performed for continuous variables wherever appropriate, while chi-square or Fisher's exact test were applied to categorical variables. A p-value of less than 0.05 was used for the statistically significant level. Statistical analysis was performed using GraphPad Prism version 9.1.0 (216).

2. SUPPLEMENTARY RESULTS:

Predictors of acute renal pathological findings in patients with eGFR < 15 ml/min/1.7m^3:

Six patients (13%) with eGFR < 15 ml/min/1.73 m^2 had acute renal pathology on kidney biopsy and got treated with immunosuppressive medications. Several clinical, laboratory and radiologic parameters were studied to determine predictors of acute renal pathology. Serum creatinine at the time of biopsy was lower in patients with acute treatable pathology than those with advanced chronic changes (737±189 vs. 1055±536; P= 0.01). In addition, patients with treatable acute pathology were less likely to be Asians than patients with non-treatable pathology (50% vs. 83%; P=0.04). Headache was the most common presenting symptom in patients with advanced chronic

changes, while none of the patients with acute renal pathology presented with headache (41% vs. 0%; P=0.001).

3. SUPPLEMENTARY REFERENCES:

S1.Asad RA, Valson AT, Kavitha V, et al. Safety and utility of kidney biopsy in patients with estimated glomerular filtration rate < 30 ml/min/1.73 m^2. Nephrology (Carlton). 2021 Aug;26(8):659-668.

S2.Xu DM, Chen M, Zhou FD, Zhao MH. Risk Factors for Severe Bleeding Complications in Percutaneous Renal Biopsy. Am J Med Sci. 2017;353(3):230-5.

S3.Sethi S, D'Agati VD, Nast CC, et al. A proposal for standardized grading of chronic changes in native kidney biopsy specimens. Kidney Int. 2017;91(4):787-9.

4. SUPPLEMENTARY TABLES:

Supplementary Table 1: Clinical outcomes of patients with treatable acute kidney pathological findings

Variable	Patient 1*	Patient 2	Patient 3*	Patient 4	Patient 5	Patient 6*
**Age, years**	48	30	40	28	61	72
**Gender**	M	F	F	M	F	M
**Race**	Asian	Asian	Asian	Middle Eastern	African	Caucasian
**Creatinine at biopsy**	412	465	559	943	969	655
**Diagnosis**	AIN	MPGN	Crescentic IgAN	Crescentic IgAN	AIN	AIN
**Chronicity score**	0	6	9	10	2	8
**Treatment**	oral steroids	IV and oral steroids, MMF	oral steroids	oral steroids	oral steroids	oral steroids
**Dialysis in admission**	No	No	No	Yes	Yes	No
**Creatinine at discharge**	546	744	517	HD	345	570
**30-day creatinine**	N/A	HD	914	HD	102	510
**90-day creatinine**	N/A	HD	N/A	HD	126	N/A

*Travelled back to their home countries
AIN: acute interstitial nephritis, F: female, IgAN: Immunoglobulin A nephropathy, HD: Hemodialysis, MMF: mycophenolate mofetil, M: male, MPGN: membranoproliferative glomerulonephritis, N/A: not available

Supplementary Table 2: Predictors of severe chronic changes in patients with eGFR < 15 ml/min per 1.73 m^2

Variable	Chronicity score ≥ 8 (n=22)	Chronicity score <8 (n=25)	P value
**Age, years**	32 (28-42)	34 (30-45)	0.62
**Male gender, n (%)**	16 (73)	17 (68)	0.72
**Race, n (%):**			
**Middle Eastern**	2 (9)	2 (8)	
**Asian**	17 (77)	20 (80)	0.74
**African**	2 (9)	3 (12)	
**Others**	1 (5)	0	
**Presenting Symptoms, n (%):**			
**Headache**	9 (41)	8 (32)	0.76
**Nausea/vomiting/Abdominal pain**	2 (9)	12 (48)	0.005
**Lower extremity edema**	3 (14)	5 (20)	0.70
**Dyspnea**	6 (27)	2 (8)	0.12
**Asymptomatic/Referred**	1 (5)	2 (8)	1.00
**History of Diabetes, n (%)**	2 (9)	4 (16)	0.67
**History of Hypertension, n (%)**	12 (55)	12(48)	0.77
**SBP on presentation, mm Hg**	169 (151-192)	172 (162-190)	0.58
**DBP on presentation, mm Hg**	101 (86-110)	105 (99-124)	0.21
**Dialysis before biopsy, n (%)**	14 (64)	11 (44)	0.24
**Creatinine at biopsy, umol/L**	890 (725 -1652)	767 (562 -1240)	0.16
**eGFR at biopsy, ml/min per 1.73 m^2**	7 (3-7)	7 (4-10)	0.29
**BUN at biopsy, mmol/L**	18 (14-24)	17 (14-22)	0.45
**Hemoglobin, g/dL**	8 (8-9)	9 (8-11)	0.07
**Calcium, mmol/L**	2.3 (2.2-2.4)	2.2 (2.1 -2.3)	0.09
**Phosphorus, mmol/L**	2.2 (1.8-2.4)	2.0 (1.6-2.3)	0.17
**PTH, pg/mL**	395 (299 -514)	505 (253 - 813)	0.18
**Albumin, g/L**	24 (22-29)	27 (26-29)	0.30
**C3, mg/dL**	95 (83-112)	103 (84-124)	0.25
**C4, mg/dL**	33 (28-38)	33 (27-41)	0.74
**UPC, mg/mmol**	500 (313-767)	586 (333-804)	0.53
**Proteinuria > 3.5 g**	16 (73)	19 (76)	1.00
**Hematuria, n (%)**	14 (64)	16 (64)	1.00
**Kidney length, mm**	96 (91-100)	98 (93-105)	0.23
**Kidney length per height (KL/H)**	0.6 (0.55 - 0.6)	0.6 (0.55-0.7)	0.25
**Cortical thickness, mm**	5 (5-7)	6 (5-7)	0.89

**Parenchymal thickness, mm**	14 (12-16)	14 (11-16)	0.92
**Kidney hyperechogenicity, n (%)**	21 (96)	22 (88)	0.36
**Corticomedullary differentiation, n (%):**<br>**Maintained**<br>**Poor**<br>**Lost**	 0<br>9 (41)<br>13 (59)	 3 (12)<br>9 (36)<br>13 (52)	0.24

Continuous variables are summarized as median (interquartile). C3: complement factor 3, C4, complement factor 4, PTH: parathyroid hormone, UPC: urine protein to creatinine ratio, DBP: diastolic blood pressure, eGFR: glomerular filtration rate, SBP: systolic blood pressure.

Supplementary Table 3: Kidney biopsy complications in patients with eGFR <15 ml/min per 1.73 m^2

Variable	Complication (n=13)	No Complication (n=34)	P value
**Age, years**	30 (27-42)	35 (30-45)	0.20
**Male gender, n (%)**	7 (54)	26 (77)	0.16
**History of diabetes, n (%)**	1 (8)	5 (15)	1.00
**History of hypertension, n (%)**	5 (39)	19 (56)	0.34
**SBP on presentation, mm Hg**	172 (159-205)	169 (152-186)	0.19
**DBP on presentation, mm Hg**	108 (102-116)	102 (90-111)	0.52
**Dialysis before biopsy, n (%)**	10 (77)	15 (44)	0.06
**Creatinine at biopsy, µmol/L**	699 (548-798)	634 (498-758)	0.22
**BUN at biopsy, mmol/L**	177 (14-25)	17 (14-23)	0.52
**Hemoglobin, g/dL**	8 (7-9)	9 (8-10)	0.02
**Calcium, mmol/L**	2.2 (2.1-2.4)	2.3 (2.2 -2.4)	0.99
**Phosphorus, mmol/L**	2.3 (1.9-2.7)	2.1 (1.6-2.3)	0.47
**PTH, pg/mL**	443 (278 -801)	405 (242-678)	0.32
**Albumin, g/L**	28 (23-29)	26 (23-29)	0.41
**Platelets, X 10^3/µL**	162 (140-245)	234 (198-301)	0.07
**PT, seconds**	11 (9-12)	10 (9.9-11)	0.98
**PTT, seconds**	29 (24-31)	28 (26-31)	0.56
**INR**	1.1 (1-1.1)	1 (0.9-1)	0.10
**UPC, mg/mmol**	630 (293-980)	522 (312-738)	0.24
**Proteinuria > 3.5 g**	10 (77)	25 (74)	1.00
**Kidney length, mm**	98 (93-103)	97 (92-103)	0.55
**Kidney length per height (KL/H)**	0.6 (0.5-0.6)	0.6 (0.5-0.6)	0.46
**Cortical thickness, mm**	6 (5-8)	5 (5-6)	0.08
**Parenchymal thickness, mm**	14 (12-16)	14 (11-16)	0.98
**Desmopressin prior to biopsy, n (%)**	5 (39)	8 (24)	0.47
**Chronicity score, n (%)**	8 (7-10)	6 (7-10)	0.43

Continuous variables are summarized as median (interquartile). C3: complement factor 3, C4, complement factor 4, PTH: parathyroid hormone, UPC: urine protein to creatinine ratio, DBP: diastolic blood pressure, eGFR: glomerular filtration rate, SBP: systolic blood pressure.

5. SUPPLEMENTARY FIGURE LEGENDS:

Supplementary Figure 1: Pie chart of the causes of advanced chronic kidney disease in patients with eGFR < 15 ml/min per 1.73 m^2. The etiology of advanced kidney disease was unknown in up to one-third of the cases. IgAN and hypertensive nephrosclerosis were the most identified causes of advanced chronic kidney disease.

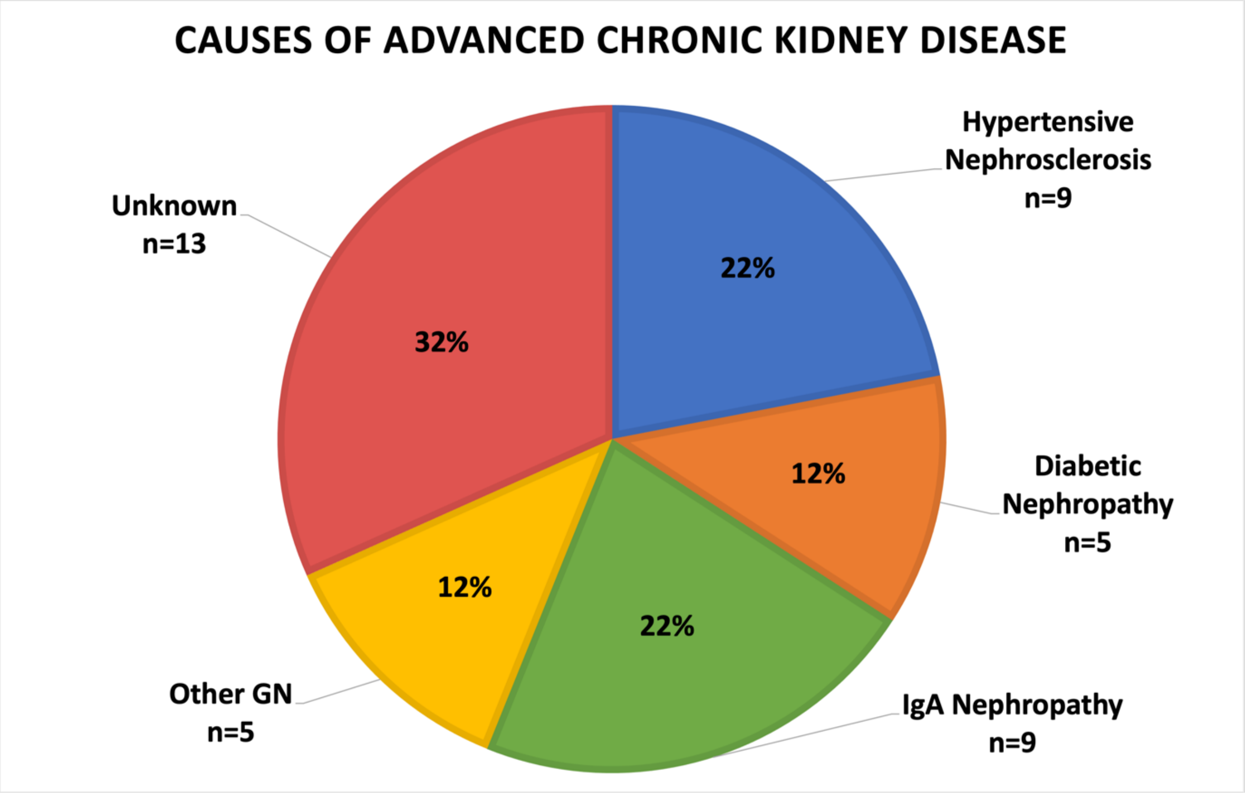

Supplement: Supplementary File (PDF) [file mmc1.pdf]
